# A Text Message–Based Intervention Targeting Alcohol Consumption Among University Students: User Satisfaction and Acceptability Study

**DOI:** 10.2196/humanfactors.9641

**Published:** 2018-07-10

**Authors:** Ulrika Müssener, Kristin Thomas, Catharina Linderoth, Matti Leijon, Marcus Bendtsen

**Affiliations:** ^1^ Department of Medical and Health Sciences Linköping University Linköping Sweden

**Keywords:** students, text messages, mobile phones, SMS, alcohol consumption intervention

## Abstract

**Background:**

Heavy consumption of alcohol among university students is a global problem, with excessive drinking being the social norm. Students can be a difficult target group to reach, and only a minority seek alcohol-related support. It is important to develop interventions that can reach university students in a way that does not further stretch the resources of the health services. Text messaging (short message service, SMS)–based interventions can enable continuous, real-time, cost-effective, brief support in a real-world setting, but there is a limited amount of evidence for effective interventions on alcohol consumption among young people based on text messaging. To address this, a text messaging–based alcohol consumption intervention, the Amadeus 3 intervention, was developed.

**Objective:**

This study explored self-reported changes in drinking habits in an intervention group and a control group. Additionally, user satisfaction among the intervention group and the experience of being allocated to a control group were explored.

**Methods:**

Students allocated to the intervention group (n=460) were asked about their drinking habits and offered the opportunity to give their opinion on the structure and content of the intervention. Students in the control group (n=436) were asked about their drinking habits and their experience in being allocated to the control group. Participants received an email containing an electronic link to a short questionnaire. Descriptive analyses of the distribution of the responses to the 12 questions for the intervention group and 5 questions for the control group were performed.

**Results:**

The response rate for the user feedback questionnaire of the intervention group was 38% (176/460) and of the control group was 30% (129/436). The variation in the content of the text messages from facts to motivational and practical advice was appreciated by 77% (135/176) participants, and 55% (97/176) found the number of messages per week to be adequate. Overall, 81% (142/176) participants stated that they had read all or nearly all the messages, and 52% (91/176) participants stated that they were drinking less, and increased awareness regarding negative consequences was expressed as the main reason for reduced alcohol consumption. Among the participants in the control group, 40% (52/129) stated that it did not matter that they had to wait for access to the intervention. Regarding actions taken while waiting for access, 48% (62/129) participants claimed that they continued to drink as before, whereas 35% (45/129) tried to reduce their consumption without any support.

**Conclusions:**

Although the main randomized controlled trial was not able to detect a statistically significant effect of the intervention, most participants in this qualitative follow-up study stated that participation in the study helped them reflect upon their consumption, leading to altered drinking habits and reduced alcohol consumption.

**Trial Registration:**

International Standard Randomized Controlled Trial Number ISRCTN95054707; http://www.isrctn.com/ISRCTN95054707 (Archived by WebCite at http://www.webcitation.org/705putNZT)

## Introduction

A large proportion of the global burden of disease is due to excessive alcohol consumption. Alcohol-related deaths increased by 30%, or approximately 5 million, between 1990 and 2010 [1]. Despite these health risks, heavy alcohol consumption among university students remains a global problem, with excessive drinking being the social norm [2,3]. In addition, research shows that students can be a difficult target group to reach, and only a minority seek alcohol-related support. Typically, local on-site student health services are commissioned to offer preventative services as well as advice and support to students who wish to reduce or discontinue drinking. However, these student health services must do so with limited resources [4]. Thus, it is important to develop interventions that can reach university students in a way that does not further stretch the resources of the health services.

Research has shown that interventions delivered by text messaging, also known as short message service (SMS), is a cost-effective method to support behavioral change [5] such as weight loss, smoking cessation, and diabetes management [6,7]. For instance, a 12-week text messaging–based intervention targeting heavy drinking among young adults was found to influence the number of days of heavy drinking and the number of drinks per drinking day [8].

Moreover, positive evidence regarding usability and user experience of text messaging–based interventions has been observed. For instance, text messages have been shown to be highly accessible to users in the sense that messages are likely to be read within minutes of being received, and interventions have been shown to be user-friendly as reading text messages requires limited time and effort [9-11]. Thus, text messaging–based interventions can enable continuous, real-time, brief support in a real-world setting [9,12,13].

This study builds on an earlier randomized controlled trial [14,15] that aimed to show the effect of using a text messaging–based alcohol consumption intervention among university students. This study has three aims:

1. To explore self-reported changes in drinking habits in an intervention group and a control group;
2. To explore user satisfaction among the intervention group given access to the novel intervention;
3. To explore the experience of being allocated to the control group.

## Methods

Ethical approval for this randomized controlled trial (RCT, ISRCTN95054707) was given by the Regional Ethical Committee in Linköping, Sweden (dnr 2016/134-31).

### Short Description of the Amadeus 3 Intervention

The Amadeus 3 intervention was developed using formative methods, including focus groups with students, an expert panel with students and professionals, and behavioral change technique analysis. The development of the program has been previously described [16]. The intervention included facts about the negative consequences of alcohol, tips on behavioral change strategies, and activities such as saying no to alcohol.

The intervention consisted of a 6-week program with a total of 62 messages. At the start, users were asked to set a goal of how much they would like to reduce their drinking. The first 4 weeks of the program had a higher frequency of 9 messages each week, followed by 7 messages in week 5, and 5 messages in week 6, all together 48 messages. Messages were sent at various times around midday, late afternoon, or early evening. Of the 62 messages, 48 were unique and 14 messages were repeated. Two messages were repeated at the start of each week, as students were asked to report via a text the number of drinks they had consumed the previous week. Following their response, they received a second text including feedback on their performance in relation to the goal they set at the start of the intervention. These paired messages were repeated every Sunday. The content of the unique messages was primarily based on information or behavioral practice. Information-based messages typically included facts about alcohol and health, consequences of excessive drinking, or tips on behavioral change strategies. Behavioral practice-based messages included asking students to reflect on or practice behavioral change; for instance, students were asked to reflect on triggers for excessive drinking or practicing saying no to drinking on a night out [16].

The messages were sent from a GSM modem and administered from a technical platform that was developed and owned by one of the authors (MB). Sending of all text messages was fully automated using this platform.

### Study Population and Recruitment

University students participating in the main Amadeus 3 study were invited to give feedback after completing the 6-week intervention and participating in the formal follow-up of the RCT [15]. Participants were recruited from 13 colleges and universities in Sweden. A total of 460 participants were allocated to the intervention group and 436 to the control group, which was offered treatment as usual (eg, other support provided at the universities such as advice and support from student health care). In the main study, follow-up data on the primary outcome were collected from 423 participants (92%) of the intervention group and 392 participants (90%) of the control group. Two reminders to complete the follow-up questionnaire were sent by email at 1 and 2 weeks after the initial request. Nonresponders were then sent text message reminders every other day for 6 days (a total of 3 text messages), and finally were contacted by phone (with a maximum of 10 calls).

After the follow-up procedure of the RCT, a second questionnaire was sent to both groups. The intervention group was asked about drinking habits and offered the opportunity to give their opinion on the structure and content of the intervention. The control group was asked about drinking habits and their experience in being allocated to the control group. The questionnaire was sent by email with 2 weekly reminders.

### Questionnaire

The intervention group was asked 12 questions. Each question had 2–7 fixed-response options with an optional free-text comment field, except for question 2 for which only a free-text comment was offered. Free-text comments gave participants an opportunity to describe other factors of importance not covered by the fixed-response options.

Initially, drinking habits were explored by 2 questions: (1) change in drinking habits during participation in the program (response options: I drink more, I drink less, I drink the same amount as before, I stopped drinking, I don’t know) and (2) possible reasons for having stopped drinking or drinking less if applicable (only free-text comment).

Experiences with the structure of the intervention were explored by 5 questions: (1) defining a goal for weekly consumption at the beginning of the program (response options: very good/good/neither good nor bad/bad/very bad/don’t know), (2) the mix of motivating, supporting, and factual content (response options: very good/good/neither good nor bad/bad/very bad/I don’t know), (3) how the participants experienced the duration of the intervention (response options: far too long /somewhat too long/just right/somewhat too short/too short/don’t know), (4) how the participants experienced the number of messages per week (response options: far too many/somewhat too many/just right/somewhat too few/too few/don’t know), and (5) how long after receiving the messages did the participants actually read them (response options: immediately/within 1 hour/within a couple of hours/same day/next day).

Experience with the content of the intervention was explored by 5 questions: (1) the content of the messages (response options: very good/good/neither good nor bad/bad/don’t know), (2) the proportion of the messages that the participant perceived to be useful (response options: all/nearly all/about half/some/nearly none/none/don’t know), (3) the proportion of all messages that were read (response options: all/nearly all/about half/some/nearly none/none/don’t know), (4) whether the participant would recommend the intervention to a friend who should reduce alcohol consumption (response options: yes/unsure/no/don’t know), and (5) whether the participant had used any additional support during the intervention (response options: no/yes).

Participants from the control group were asked 5 questions. Each question had 4 or 5 fixed-response options and offered an optional free-text comment field, except for question 2 for which only a free-text comment was provided.

Drinking habits of the control group were explored by the same 2 questions as for the intervention group: (1) change in drinking habits since the beginning of the trial (response options: I drink more, I drink less, I drink the same amount as before, I stopped drinking, I don’t know) and (2) possible reasons for having stopped drinking or drinking less if applicable (only free-text comment).

Experience with and actions taken from being randomized to the control group were explored by 2 questions: (1) experience of having to wait for support from the program (response options: disappointed because I expected to get support immediately/ok because I had time to reflect upon my alcohol habits/didn’t matter/don’t know) and (2) actions taken while waiting for support from the program (response options: I used other support [type of support was to be specified]/I decided to reduce my consumption until I got support from the program/I tried to reduce my consumption without support/I continued to drink as before/don’t know). The final question explored whether control group participants felt that the information regarding the study design was sufficient when signing up (response options: yes, very good/yes, ok/no I lacked information [type of information was to be specified]/don’t know).

### Data Analysis

Descriptive analyses of the distribution of the responses to the 12 questions for the intervention group and 5 questions for the control group were performed. In the first step of the analyses, all free-text comments to each question were read by 2 authors (UM and CL). In the second step, UM chose a variety of the most crucial free-text comments for each question. In the third step of the analysis, UM presented the chosen comments to the other authors and, after discussion, the comments that captured the main content of the specific question regarding the aim of the study were chosen. The free-text comments were used to underscore and illustrate the pattern of responses to the fixed-response options. The number after each comment represented the individual code that was assigned to each of the respondents. “/.../” showed that part of the free-text comment had been omitted.

## Results

### Overview

Baseline data were used to assess differences between responders and nonresponders. Variables included sex, age, marital status, total number of standard drinks consumed per week, number of episodes of heavy drinking, highest estimated blood alcohol concentration, and the number of negative consequences experienced. The response rate for the intervention group was 38.2% (176/460). As can be seen in Table 1, the baseline characteristics of responders were similar to those of the nonresponders except for sex; the data indicated that females were over-represented in the responders group. The response rate for the control group was 29.6% (129/436). No significant differences were found among participants and nonparticipants with respect to baseline characteristics (Table 2).

**Table 1:** Baseline characteristics of the responders and nonresponders in the intervention group follow-up.

Variable		Nonresponders (n=284)	Responders (n=176)	*P* value
Sex, n (% female)		157 (55.3)	108 (61.4)	.001^a^
Age (years), median (IQR^b^)		24 (5.5)	24 (3.5)	NS^c^
Marital status, n (% single)		182 (64.1)	106 (60.2)	NS
Total weekly alcohol consumption (in standard drinks), median (IQR)		12 (10)	12 (8.25)	NS
**Heavy episodic drinking, n (%)**				NS
	Two or three times a month	79 (27.8)	32 (18.2)	
	Once or twice a week	149 (52.5)	92 (52.3)	
	Three times or more a week	56 (19.7)	52 (29.5)	
eBAC^d^, median (IQR)		1.37 (1.11)	1.32 (1.12)	NS
Number of negative consequences, median (IQR)		3 (3)	3 (3)	NS

^a^Pearson’s chi-square test indicated that the distributions in the 2 groups differed significantly (*χ*^2^_1_=10.652, *P*=.001).

^b^IQR: interquartile range.

^c^NS: not significant.

^d^eBAC: estimated blood alcohol concentration.

**Table 2:** Baseline characteristics of the responders and nonresponders in the control group follow-up.

Variables		Nonresponders (n=307)		Responders (n=129)		*P* value	
Sex, n (% female)		172 (56.0)		72 (55.8)		NS^a^	
Age (years), median (IQR^b^)		24 (6)		23 (5)		NS	
Marital status, n (% single)		185 (60.3)		71 (55.0)		NS	
Total weekly alcohol consumption (in standard drinks), median (IQR)		12 (10)		11 (8)		NS	
**Heavy episodic drinking, n (%)**						NS	
	Two or three times a month		87 (28.3)		43 (33.3)		
	Once or twice a week		150 (48.9)		68 (52.7)		
	Three times or more a week		70 (22.8)		18 (14.0)		
eBAC^c^, median (IQR)		1.48 (1.22)		1.33 (1.35)		NS	
Number of negative consequences, median (IQR)		3 (3)		2 (3)		NS	

^a^NS: not significant.

^b^IQR: interquartile range.

^c^eBAC: estimated blood alcohol concentration.

In the intervention group, 70.4% (124/176) of the participants provided at least one comment to the 12 questions and the other 29.5% (52/176) did not offer any additional comments. In the intervention group, 54.5% (96/176) participants provided comments on possible reasons for having stopped drinking or drinking less, 15.9% (28/176) on the question about change in drinking habits during the program, and 2.3% (4/176) on the question on the time between receiving and reading the messages. On average, approximately 20 comments were provided for each question.

In the control group, 50.3% (65/129) of the participants provided comments to the 5 questions, and the other 49.6% (64/129) did not offer any additional comments. As in the intervention group, most comments were on possible reasons for having stopped drinking or drinking less (42.6%, 55/129) and the question on changes in drinking habits during the program (7.0%, 9/129). The fewest number of comments was provided for the question on whether the participants found that the information regarding the study design was sufficient when signing up 4.6% (6/129). On average, around 17 comments were provided for each question.

We report the responses to the relevant questions and include citations from the free-text comments for each heading.

### Changes in Drinking Habits and Reasons for Reduced Consumption

#### Intervention Group

In the intervention group, 34.6% (61/176) of participants reported that they consumed the same amount of alcohol as that before the intervention, 51.7% (91/176) stated that they were drinking less, 4.5% (8/176) stated that they had stopped drinking altogether, 4.0% (7/176) stated that they were drinking more than before, and 5.1% (9/176) answered that they did not know. In the free-text comments, some participants expressed that participation in the study helped them reflect on their alcohol consumption, leading to changed drinking habits.

I don’t want to stop drinking at a party, but I reflect more on my consumption since I started participating in the study. So participation has probably affected me positively

2540432

I reflect about my consumption more. I have fewer binges, but I still drink far too much at times and get memory loss.

254551

Not the greatest change, but for example, I don’t drink at home before the party any more.

2545551

Increased awareness of negative consequences was expressed as the main reason for reduced alcohol consumption among participants in the intervention group who reduced or stopped drinking during the intervention. In their comments, many described having experienced consequences regarding economics, health, relationships, and exam results.

I’m more aware of the negative consequences that come with drinking and for me that means that I now want to start living a healthier life.

2540768

I think more about what alcohol does to my health, the economy, relationships, work, etc

2540888

I reflect more about my drinking nowadays. And I think more about how much I drank or how much I planned to drink and how that affects me. Thus, I sometimes completely refrain from alcohol or choose to drink less when something is to be celebrated. I received a lot of good advice from the study.

2540874

Increased motivation, loss of control when drinking, and emotional consequences of alcohol consumption were also mentioned in the free-text comments.

Motivation. /.../ I say and do things that I’m ashamed of the day after drinking. I get anxiety and I get very sick the day after.

2568730

Because I don’t like to get these memory shutters. What made me think was when you had to count the units of alcohol and I saw how much I drank.

2537333

Some participants also described their reduction in alcohol consumption because of life changes, such as getting pregnant, moving to another city, entering working life, as well as new social networks and relationships.

Change of habits because of a new job.

2540674

Changed living situation, I don’t live in a student city any longer.

2541284

#### Control Group

Of the participants in the control group, 41.1% (53/129) reported that they drank the same amount as that before the trial, 32.5% (42/129) stated that they were drinking less, 1.5% (2/129) stated that they had stopped drinking alcohol, 11.6% (15/129) stated that they drank more than before, and 13.2% (17/129) answered that they did not know. The reasons for reduced consumption seem to be similar to those of the intervention group, such as awareness of their drinking habits, which in turn affected changes in lifestyle.

I don’t hang out with the same people anymore /.../ I don’t have the same drinking habits that students in Norrkoping have

2154339

I feel better. I have a healthier relationship with alcohol. I live together with my partner now. I focus more on my studies, training, and health.

2539339

### Satisfaction With the Structure of the Intervention

The intervention began with a request to all participants in the intervention group to define a goal for their drinking habits. Of the participants, 77.8% (137/176) agreed that having to define a goal was good or very good at the beginning of the intervention.

I think it was good because you always thought about that goal. Once you drank, you thought about exactly how much you drank, which caused you to drink less.

2540464

Well, it made me feel guilty when I answered the text messages and wrote how much I had been drinking this week.

2568862

On the other hand, 2.3% (4/176) of the participants reported that setting a goal was bad, and one participant commented that it created an expectation from the participant and therefore had the opposite effect.

I felt concerned about the demands or expectations, and it had the opposite effect

2541027

The variation in the content of the text messages from facts to motivational and practical advice was appreciated by 76.7% of the participants (135/176), particularly among those who reduced or quit drinking. Some participants emphasized the need for different content because people have different needs; some wanted more facts, and 34.6% (61/176) said the variation was bad or very bad.

A good variety in order to cover the different areas that may cause problems.

2540491

People do handle things differently - then variety is good.

2544151

I liked when there were facts, eg, how alcohol affect sleep. Even more facts like that would be good!

2546086

The duration of the 6-week intervention was perceived to be adequate by 55.1% of the participants (97/176).

During the program, I thought it was an unnecessarily long number of weeks and not very fulfilling. It was only afterward that I began to think that it was quite worthwhile. I think the length of it was the most rewarding aspect for me, to actively reflect on alcohol for a relatively long time.

2543442

Of the participants, 17.0% (30/176) stated that it was too short, and 19.9% (35/176) stated that it was too long. One participant suggested sending less than one text message per day.

No problem with 6 weeks, but maybe not with text messages every day. It felt almost tedious. For me, the problem arose at the weekends when there was a party, and that is the time when you might need the text messages as a reminder.

2541735

Of the participants, 54.5% (96/176) found the number of messages per week to be about right; 34.1% (60/176) thought there were too many messages. One participant said that they looked forward to the daily text message, but that there was a lot of information to digest.

Every day I looked forward to the text messages, but on the other hand it was very extensive /.../there was a lot of information to take in

2540677

On average, 19.9% (35/176) of participants read the text messages immediately when they received them, 53.4% (94/176) read the messages within 1 hour, 15.3% (27/176) read the messages within a couple of hours, and 2.3% (4/176) of participants read the text messages the next day.

### Satisfaction With the Content of the Intervention

Regarding the content of the text messages, 64.2% of participants (113/176) in the intervention group found the content good or very good. Among those who were in favor of the content, some emphasized that the messages changed how they thought about alcohol consumption, and they were reminded about why they wanted to reduce or stop drinking. Others stated that the messages made them reflect on their drinking in a more conscious way.

Interesting information. I had expected horror propaganda, but many of the issues were about putting your alcohol problems in perspective and reflecting on your habits

2540667

Of the participants, 6.2% (11/176) found the content bad or very bad. Some participants who were still drinking as before perceived the messages as irritating and impersonal. One participant described the messages as a bad joke.

Aggravating. It was like a bad joke, obvious and impersonal. But the program gave us good laughs at our party anyway. I thought it was so bad that I ended in advance.

2544204

A total of 27.8% (49/176) did not find the content either good or bad. Some emphasized that the actual content of the messages was not very important; rather, it was more important to be reminded and encouraged to think about one’s alcohol consumption.

I did not feel like it was the content itself that mattered, many of the text messages repeated things I already know. For me what made the difference, however, was being reminded to be aware of my plans for drinking - which I did by reading the text messages. What was in the messages did not matter. And, it felt like someone was supervising a little when the messages came, which also made me less motivated to drink.

2539338

Some thought that the messages were interesting and relevant initially but then became repetitive, and they suggested shorter messages less often and that the messages should be sent out earlier in the evenings, before they started to drink. More supporting and motivating messages before weekends were appreciated.

You had the chance to analyze your decision to drink, so sometimes you chose not to drink alcohol because you were affected by the message.

2543929

The proportion of messages that the participants perceived to be useful differed. Only 35.8% of participants (63/176) thought that all or nearly all messages were useful for their situation. Some experienced that the messages gave a feeling that somebody cared.

I was thankful for the messages in any case. It felt like someone cared, although virtual.

2540886

Among those who were still drinking the same amount as before the intervention and who estimated that none of the messages were useful, one participant said that the messages had the opposite effect: a desire to drink.

Seemed that the messages were far too goading and made me think of alcohol more and to drink more, the opposite of their purpose

2541098

In all, 80.7% of participants (142/176) stated that they had read all or nearly all the messages and only 7.9% of participants (14/176) reported that they only read a few or none of the messages. Furthermore, 48.3% of the participants (85/176) said that they would recommend the intervention to a friend, 39.2% (69/176) said that they were unsure or did not know if they would recommend the intervention, and 12.5% (22/176) would not recommend it.

Uncertain. If one really needs help, more action is required. But it can be a good way to reflect on ones’ drinking as well as being reminded.

2540432

Yes, but only if it's a person who has previously thought about reducing his or her alcohol consumption. For a person who had not reflected on it earlier, the program would probably not be so useful.

2540768

Of the participants, 95.4% (168/176) had not used additional support during the intervention. Seven free-text comments mentioned the use of the following additional support: reading a book regarding the power of habits, face-to-face encounters with professionals, dialog with relatives, medication, and therapy.

### Experiences of Being Randomized Into the Control Group

Among the participants in the control group, 40.3% (52/129) expressed that it did not matter that they had to wait for access to the intervention, and 20.9% (27/129) stated that it was fine to wait because it gave them time to reflect on their consumption, whereas 27.9% of participants (36/129) expressed disappointment with having to wait for support.

Of course, I wanted to get the support as quickly as possible, but there were no big problems having to wait.

546113

However, having to wait seemed a bit frustrating at first.

2544776

Regarding actions taken while waiting for access, 48.1% of the participants (62/129) claimed that they continued to drink as before, and 34.9% (45/129) tried to reduce their consumption without any support; 3.1% of participants (4/129) reported that they decided to wait until they were given access to the intervention, and the same number of participants reported that they used other aids, such as medication and support from the alcohol-dependence units.

The final question explored whether the participants in the control group found that the information regarding the study design was sufficient when signing up. Of the participants, 67.4% (87/129) stated that the information was good or very good, 27.1% (35/129) answered that they did not know, and 5.4% (7/129) stated that they did not think that the information was sufficient.

You did not really know what you were getting yourself into; the study was quite unclear and it was hard to know when the 3 months had passed or were still ongoing

2540443

## Discussion

### Principal Findings

The main findings of this study are that most of the participants in the intervention group state that participation in the study helps them to reflect on their consumption, leading to altered drinking habits and reduced alcohol consumption. Most of the participants appreciate the variation in the content of the text messages from facts to motivational and practical advice. The results also shine light on the experience of being allocated to a control group and that it does not matter that they must wait for access to the intervention.

Despite the low response rate of 34%, participants in this user evaluation study provided valuable information regarding alcohol consumption and changes in drinking habits, as well as user satisfaction and the experience of being allocated to a control group that can be used in further work regarding alcohol-related support. A note of caution, however, should be made. There is an over-representation of females who responded in the intervention group (Table 1). Apart from this, participants are broadly representative of the study population (participants in the RCT).

Many individuals find the support helpful, yet in terms of being effective toward a general nontreatment-seeking population, there is more work to be done. The reasons given for reduced consumption seem to be the same in both the control and intervention groups: increased awareness regarding negative consequences for health, economics, relationships, study results, and work, as well as changes in civil status. However, it is not possible to say why the reasons are the same in both groups. New interventions need to focus on the factors that both groups estimate to be important when it comes to changes in lifestyle.

Previous research shows that despite the promising potential of text messaging–based interventions, it is difficult to tell how effectiveness may be optimized through the content and structure choices [11,17]. This study sheds light on some of these questions because the results show that the overall structure and content of the text messaging–based intervention are well received by most participants, regardless of whether they reduce their drinking. Most agree that the variation is valuable, particularly among those who reduced or stopped drinking. One possible conclusion, also noted in previous research using a text messaging–based intervention among students who smoked, is that text messaging–based cessation interventions are more suitable for those who are motivated to use these types of programs, and those who are not fully motivated or determined to change their lifestyle habits may find other types of support more suitable, for example face-to-face meetings with professionals [18].

Participants in the intervention group who appreciated the content stressed that the messages changed their thinking about alcohol consumption. Among participants who were less appreciative of the content, some emphasized that the content itself is of less importance. Instead, most of the gain is in being reminded and encouraged to reflect on one’s consumption. It is unclear if it is the content of the messages or the frequent reminders and reinforcement of having committed oneself to reduce one’s drinking that matters. Similar results were shown in a previous study using a text messaging–based intervention to stop smoking among young people [19], and mechanisms of the effect of this type of intervention remain to be identified and studied further. The remarks that the same messages are perceived as irritating, impersonal, and repetitive by some and useful by others reflect the limitations imposed by untailored interventions and highlight the difficulty in developing an intervention that fits all, an issue that has been discussed extensively [20]. The variation in content is appreciated by most participants, but the proportion of messages that the participants perceived to be useful differs. The feeling of being cared for is mentioned as important among those who think that the messages are valuable to their situation.

Additional support is used by few, and because the intervention should be a complement to other support provided at the universities, the results are not affected in any direction. Previous research shows that only a minority of students seek advice and support from student health care, and our results emphasized the need to further develop new means of reaching students who drink excessively [21].

Being randomized into a control group implies having to wait for support for approximately 3 months, but 40.3% of the participants (52/129) expressed that delayed support did not matter. Some used the waiting time to reflect on their consumption. Concern regarding the ethics of assigning participants actively ready for change to control groups has been raised [22] and asking participants in control groups to wait to seek treatment may lessen their natural help-seeking behaviors [23]. However, participants in this study were free to seek other treatments, but only a few chose to do so; indeed, half of the participants continued to drink as before.

The strengths of this study are that participants were recruited from 14 colleges and universities in Sweden and that there were many free-text comments to most of the questions. The intervention is fully automated and did not require the user to remember to log in to a web portal or similar website throughout the intervention.

### Limitations

Limitations of the study include the low response rate and the relatively short questionnaires used to explore the views of the participants. The duration of the intervention was adequate for only about half of the participants, indicating that the optimal duration of the intervention is still to be established.

Several steps have been taken to ensure the validity of the results. Two authors read the free-text comments independently many times. The first author selected a variety of the most crucial free-text comments, and then the chosen free-text comments were presented and discussed with the other authors, and comments that captured the main content of the specific question about the aim of the study were chosen. Free-text comments not agreed on by all authors were excluded.

### Conclusions

Reflecting on alcohol consumption may help young people change their drinking habits and reduce their alcohol consumption. Variation in the content of the intervention from facts to motivational and practical advice seems to be satisfactory, but the optimal duration of the intervention, as well as the number of messages per week, is still to be established. Further work is needed to determine what aspects matter to support students who wish to reduce or quit drinking. To obtain such knowledge, students’ experiences are probably highly significant, especially in the context of improving understanding of the mechanisms behind a successful text messaging–based intervention. Deeper knowledge is needed about whether it is the content itself that is important or if the gain is in being reminded frequently and encouraged to reflect on one’s consumption.

## Abbreviations

eBAC: estimated blood alcohol concentration
IQR: interquartile range
NS: not significant
RCT: randomized clinical trial
SMS: short message service
